# Schottky barrier at graphene/metal oxide interfaces: insight from first-principles calculations

**DOI:** 10.1038/srep41771

**Published:** 2017-02-06

**Authors:** Kai Cheng, Nannan Han, Yan Su, Junfeng Zhang, Jijun Zhao

**Affiliations:** 1 Key Laboratory of Materials Modification by Laser, Ion and Electron Beams (Dalian University of Technology), Dalian 116024, P. R. China; 2 School of physics and information Engineering (Shanxi Normal University), Linfen 041000, P. R. China

## Abstract

Anode materials play an important role in determining the performance of lithium ion batteries. In experiment, graphene (GR)/metal oxide (MO) composites possess excellent electrochemical properties and are promising anode materials. Here we perform density functional theory calculations to explore the interfacial interaction between GR and MO. Our result reveals generally weak physical interactions between GR and several MOs (including Cu2O, NiO). The Schottky barrier height (SBH) in these metal/semiconductor heterostructures are computed using the macroscopically averaged electrostatic potential method, and the role of interfacial dipole is discussed. The calculated SBHs below 1 eV suggest low contact resistance; thus these GR/MO composites are favorable anode materials for better lithium ion batteries.

The wide usage of lithium ion batteries (LIBs) in the portable electronic devices and electric vehicles require high energy density, fast charge/discharge rate, and long cycling lifespan^1^. Anode materials play an important role in determining the performance of LIBs. Currently, graphite is the major commercial anode material, but it suffers from a limited theoretical capacity of 372 mA h g^−1^ to meet the increasing requirement^2^. Much attention has been paid to explore better candidate anode materials, including metal oxides (MOs)^3^,^4^,^5^,^6^,^7^, metal sulfide^8^,^9^, metal nitrides^10^, carbonaceous materials^11^,^12^, lithium alloy^13^, and polymers^14^. MOs (especially in the form of nanostructures) are promising electrode materials for LIBs because of their high specific capacity up to 700 mA h g^−1^ 6. Furthermore, many MOs such as iron oxides and manganese oxides are abundant in nature and thus low cost. Unfortunately, their cycling stability cannot meet the requirements^15^,^16^ for practical application due to large volume variation during charge/discharge (Li^+^ intercalation/extraction) processes. In addition, the use of MOs also leads to poor lithium ionic transport and electronic conductivity^17^.

Parallel to the research and development of MOs, tremendous heart-stirring revolutions occur in the development of electrochemistry storage with graphene (GR) based materials. GR has not only extremely high electrical conductivity but also a big specific area of 2620 m^2 ^g^−1^, which endows more active sites for electrochemical reaction. Yoo *et al*. reported a capacity of 540 mA h g^−1^ for graphene nanosheets, while much higher capacity could be obtained by adding CNTs or fullerene into graphene layers to increase interlayer distance^11^. High-quality few-layer graphene nanosheets were reported to possess an initial capacity as high as 1264 mA h g^−1^, which was attributed to the large surface area (492.5 m^2 ^g^−1^), curled morphology, and disordered structure^18^. Though there is a satisfactory increase in capacity, these GR-based materials still face the deficiencies of large irreversible capacity, fast capacity fading (mainly due to serious agglomeration and re-stacking of GR layers) and a lack of obvious voltage plateau^19^.

To address the above issues of MOs and GR simultaneously, a successful approach is to incorporate MOs with GR into composite^19^. Typically, the GR/MO composites have several architectures: anchored model, wrapped model, encapsulated model, sandwich-like model and mixed model. The resulted GR/MOs composites not only avoid the volume change in MO anode and enhance the electric conductivity, but also weaken GR’s agglomeration due to van der Waals interactions, resulting in an excellent performance. Zhu reported that NiO particles anchored on the surface of reduced graphene has specific capacities of 1641 mA h g^−1^ and 1097 mA h g^−1^ for the first discharge and charge respectively, and the GR/NiO composite also shows a good rate capacity and cyclic performance^20^. Subsequently, Zhou *et al*. attributed the improvement of lithium storage mainly to the oxygen bridges between NiO and GR^21^. Xu *et al*. synthesized the core-shell GR/Cu_2_O composite, which delivers a reversible capacity of 458 mA h g^−1^ after 50 cycles at a current density of 100 mA h g^−1^ 22. Other GR/MO composites, including SnO_2_^23^, Co_3_O_4_^24^, Fe_2_O_3_^25^, TiO_2_^26^ also show excellent capacity and favorable cyclic performance.

To date, much attention has been paid to the capacity and cycle stable performance of GR/MO composites as electrode materials, while little is known about the interfacial properties. For a composite, an interface region exists and greatly influences its electronic properties and thus material performance. It is well known that, when two solids are joined in a heterojunction, the electronic structure is perturbed locally near the interface. At a few atomic layers away from the interface (i.e., the interior regions of the component materials on the two sides), the electronic structure would come back to that of individual solid. Therefore, the band discontinuity is of technological importance for both metal-semiconductor and semiconductor-semiconductor interfaces. The Schottky barrier height (SBH) is the key parameter for the interfacial electrical characteristics in a metal-semiconductor heterojunction^27^,^28^. Ohmic contact or Schottky contact with a low barrier means a small contact resistance, thus less power loss in the interfacial region. Most likely, the GR/MO interface forms a Schottky barrier for electron transport, which has important influence on the performance of the GR/MO composites as anode material and has not been theoretically exploited yet from within our knowledge.

In this paper, we investigate for the first time the electronic structure of several GR/MO interfaces and explain why the composite show better electrochemical performance than both GR and MOs. Then we compute their SBHs using the macroscopically averaged electrostatic potential as an intrinsic energy scale to refer to all the energies^29^. The role of interfacial dipole on the magnitude of SBH is also discussed. Our first-principle results suggest that the GR/MO composites with low SBH are favorable anode materials for better LIBs.

## Computational Methods and Models

All first-principles calculations were carried out using the Vienna Ab initio Simulation Package (VASP) based on spin-polarized density functional theory (DFT)^30^ using a plane-wave basis set. To treat the exchange-correlation interaction of electrons, we chose the Perdew-Burke-Ernzerhof (PBE) functional^31^ within the generalized gradient approximation (GGA). The electron-ion interactions were described by the projector augmented wave (PAW) potentials^32^. A dipole correction was applied to avoid spurious interactions between periodic images of the slab. To take into account the long-range van der Waals (vdW) interactions between GR and MOs, the semi-empirical dispersion-corrected DFT-D3 scheme proposed by Grimme^33^ was adopted. The energy cutoff for plane-wave basis was set at 450 eV. We used the Γ-centered **k** grids for Brillouin zone (BZ) integrations, with a uniform spacing of 2π × 0.02 Å^−1^. It is known that standard GGA methods often cannot describe the systems with localized (strongly correlated) *d* and *f* electrons, such as transition metal oxides, and even fail to predict the energy levels of *d* and *f* electrons. If the *d* orbitals are close to the valence band maximum (VBM), this deficiency needs to be corrected in order to locate the correct VBM energy. Therefore, we applied the GGA + U correction on the Ni (*d*) and Cu (*d*) states for the electronic structure calculations. We chose U = 6.2 and J = 0.98 eV for Ni^34^ and U = 6 and J = 1 eV for Cu^35^ respectively. A convergence criterion of 10^−4^ eV for the total energy and 0.02 eV/Å for the force were adopted for self-consistent calculation and geometry optimization.

**Figure 1:**
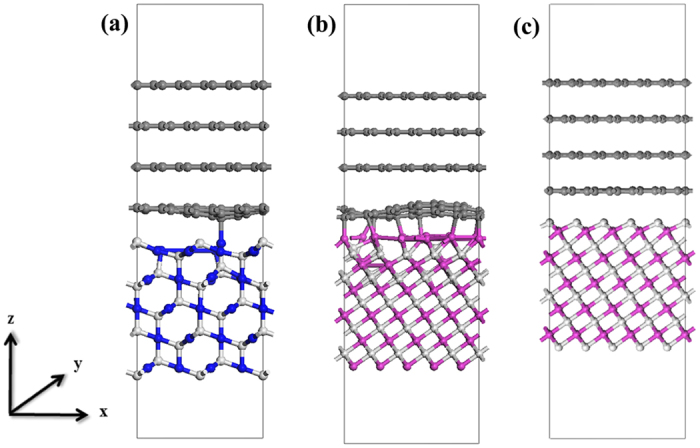
The light gray balls for O atoms and the dark gray balls for C atoms (**a**) GR/Cu_2_O-O, the blue balls for Cu atoms, (**b**) GR/NiO-Ni, the purse balls for Ni atoms, (**c**) GR/NiO-O. The interface is perpendicular to *z* axis for these heterostructures.

Interface between graphene and Cu_2_O or NiO have been considered in this paper, since the GR/MO composites with these two oxide materials show excellent performance^20^,^21^,^22^. First, we optimized the lattice parameters of bulk MO solids, and obtained the equilibrium lattice parameters of *a* = 4.269 Å for body-centered cubic Cu_2_O and *a* = 4.209 Å for face-centered cubic NiO, which agree well with the experimental values (*a* = 4.267 Å for Cu_2_O^36^, and *a* = 4.177 Å for NiO^37^). Then we constructed slab supercell models for Cu_2_O and NiO using the crystal lattice parameters, which are laterally co-periodic to the graphene with lattice mismatch less than 2% (see Table 1 for details)^38^. A previous theoretical study has shown that a small lattice mismatch ≤2% has little effect on the electronic properties of graphene^39^. To model the GR/MO heterostructure, the simulation supercell includes a slab of five layers of MO, a slab of four-layer graphene, and a vacuum region of 16 Å thick along *z* axis (see Fig. 1). Note that we used multilayer GR instead of monolayer GR to ensure the ‘bulk like’ electronic behavior at the GR side of the heterostructures. Actually, the composite anode materials prepared in experiment always include multilayer GR of typically 1~7 nm thickness^22^,^25^,^40^.

Initially, the equilibrium distance between MO slab and few-layer graphene was carefully determined by a series of single-point energy calculations. The equilibrium distance is defined as the difference of the average height of the bottom layer of GR and the top layer of MOs. We tested the distances from 2.5 to 3.5 Å with a separation of 0.1 Å, and obtained the best distances of 2.7, 2.8 and 2.9 Å for GR/Cu2O-O (Figure S1a in Supporting Information), GR/NiO-Ni (Figure S1b) and GR/NiO-O (Figure S1c), respectively. Then, based on these best distances, we optimized the heterostructures. During the geometry optimization of each MO/GR heterostructure, we fixed the three bottom layers of MO and the three layers of graphene on the other side, and only allowed the atoms closest to the interface (i.e., two MO layers plus one GR layer) to relax. We chose the close packed (111) face for each MO crystal. When cleaving NiO, we achieved a ‘O’ determinate surface and a ‘Ni’ determinate surface for NiO, respectively, while only a ‘O’ determinate surface is obtained for Cu_2_O. Therefore, here we consider a total of three different heterostructure configurations, as displayed in Fig. 1. For simplicity, in the following discussions, we name them as GR/Cu_2_O-O, GR/NiO-Ni, and GR/NiO-O, respectively.

## Results and Discussions

### Interaction between GRs and MOs

Based on the optimized structure of the three heterostructures, we investigate the interactions between GR and MOs. With full relaxation, the interfacial GR and MO layers are severely distorted in the GR/NiO-Ni heterostructure. The distortion is relatively weaker in the GR/Cu_2_O-O system, while it becomes even negligible in the GR/NiO-O interface (see Fig. 1). The strength of GR/MOs’ interactions can be characterized by the binding energy defined as


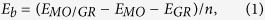


where *E*_*MO*/*GR*_ is the total energy of heterostructures; *E*_*MO*_, *E*_*GR*_ are the total energy of MO slab and four-layer GR, respectively; *n* denotes the number of C atoms in the contact GR layer. The equilibrium distance and binding energy between GR and MO parts for all heterostructures are summarized in Table 1. For GR/NiO-Ni, the equilibrium average distance is 2.5 Å and the binding energy is 0.119 eV, indicating relatively stronger interaction between GR and ‘Ni’ determinate surface of NiO. A number of C-Ni bonds are formed at the interface, which cause distortion of Ni lattice atoms at the contact layer as well as curl of the closest GR layer. For GR/NiO-O, the larger equilibrium distance (2.959 Å) and lower binding energy (0.044 eV) suggest a weaker interaction between GR and ‘O’ determinate NiO surface than GR/NiO-Ni. For GR/Cu_2_O-O, the average distance is about 2.56 Å, which can be compared to an experiment value of 2.4 Å^22^. Note that a C atom from the GR layer directly bonds with an underneath Cu atom (bond length: 2.121 Å), while all O atoms in the contact layer of Cu_2_O remain at their initial positions. Even though the C-Cu bond reduces the equilibrium average distance, the overall interaction between GR and ‘O’ determinate surface of Cu_2_O still belongs to weak physical adsorption with binding energy of 0.016 eV.

**Figure 2:**
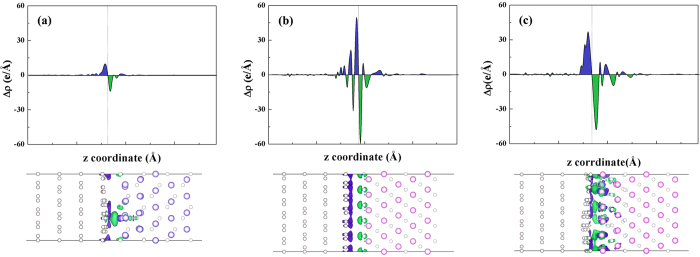
Plane-averaged electron density difference along the direction perpendicular to the interface (upper) and sides view of plots of electron density difference (lower) for (**a**) GR/Cu_2_O-O, (**b**) GR/NiO-Ni, and (**c**) GR/NiO-O. The interface is perpendicular to *z* axis for these heterostructures. Isosurface value for three structures is 0.002 e/Å^3^, where the accumulation and depletion of electrons are represented in purse and green, respectively.

To visualize the GR/MO interaction, we present the electron density differences in Fig. 2. Interfacial charges transfer from MO to GR for all three heterostructures. Clearly, charge redistribution mainly occurs at the interfacial region between the first MO and GR atom layer, in line with the fact that the gap states emerge mainly in the first layer of MOs but almost vanish in the second MOs layer. In other words, gap states do not penetrate deeply into the ‘bulk’ region of either GR or MOs. A careful examination show that the electrons in GR side extend about 0.8 Å for GR/Cu_2_O-O, 1.1 Å for GR/NiO-O, and 1.2 Å for GR/NiO-Ni, respectively, while the positive charges in MO side extend about 2.4 Å for GR/Cu_2_O, 1.9 Å for GR/NiO-O, and 1.2 Å for GR/NiO-Ni, respectively. In total, the electrons extend less distance in the GR region. The physical explanation for this phenomenon is that GR has a very high electron density. Charge transfer results in the formation of interfacial dipole. Among them, GR/Cu_2_O-O shows weakest interfacial dipole, while GR/NiO-Ni and GR/NiO-O exhibit comparable behavior. The plane-averaged electron density difference in Fig. 2(b) further shows that a small dipole is produced in GR/Cu_2_O-O, while it is much stronger in GR/NiO-Ni and GR/NiO-O. As we will discuss later, such interfacial dipole plays a significant role in the amplitude of SBH.

**Figure 3:**
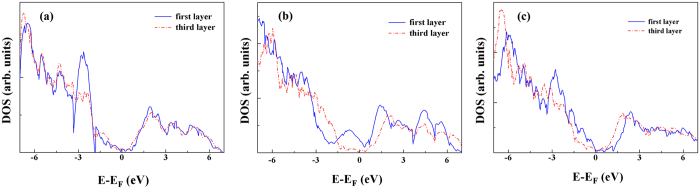
Local density of states (LDOS) of (**a**) GR/Cu_2_O-O, (**b**) GR/NiO-Ni, and (**c**) GR/NiO-O. The LDOS comes from the most bottom layer of GR and the most third bottom layer of GR.

To elucidate the modification of electronic states in the GR/MO composite with regard to the individual systems, we also calculated the local density of states (LDOS) for selected carbon atoms in three heterostructures, as shown in Fig. 3. Here the third layer is the third bottom C layer of GR in Fig. 1, which represents the pristine monolayer of GR. The first layer is the bottom layer of GR, which contacts with MO slab. For GR/Cu_2_O-O, the LDOS of the contact GR layer almost coincides with the third layer GR, except for a sudden peak that originates from the formation of a C-Cu bond mentioned above. As for GR/NiO-O, the diagram of DOS of the first layer keeps the basic shape of the third layer along with a small red shift (by about 0.4 eV) due to the existence of interfacial dipole. From the LDOS of GR/NiO-Ni, the contact GR layer shows metallic behavior, because of slightly stronger interaction as discussed above.

To briefly summarize, the small binding energies of GR/NiO-O (0.044 eV) and GR/Cu_2_O-O (0.019 eV) suggest the interactions are weak, even though a relatively larger dipole exists in the GR/NiO-O interface. The strength of these binding energies is comparable to recent theoretical results (about 0.04 eV) of the other GR-based heterostructures such as GR/phosphorene^40^, GR/Si^41^, GR/MoS_2_^42^, GR/*h*-BN^43^. This weak interaction can be enhanced by a metal layer inserted at the interfacial region^44^,^45^. But due to the relatively weak interactions between GR and ‘O’ determinate surfaces of MOs, MO materials can hybrid with GR by keeping their intrinsic electronic structures undisturbed. Thus, ‘O’ determinate MO surfaces can retain the satisfactory capacity in the GR/MO composite electrode.

### SBH of GR/MO interfaces

The determination of SBH cannot be achieved in first-principles calculations from a direct comparison of the corresponding band edges in the two components as obtained from two individual band-structure calculations. This is because the band energies are determined with respect to a macroscopic average potential in the solid^28^, while the absolute value of them is considered arbitrary in the pseudopotential calculation^46^. The macroscopic average potential (along the *z* axis) is defined as 
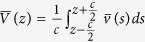
, where *c* is the lattice parameter. The 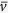 is the plane-averaged Hartree potential, which is defined as 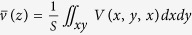, where the *S* is the lattice area of one unit cell in *x-y* plane and *V*(*x, y, x*) is the Hartree potential. Once a heterostructure is constructed, there is no internal reference level to refer to. On the other hand, formation of an interface out of two single materials leads the electron charge density to readjust in the interfacial region and the generation of a dipole in such a way as to minimize the total energy of the system. Usually, the problem of band alignment between two component solids can be divided into two parts: one part can be expressed as the difference between quantities which are intrinsic to each solid, and another part includes the effect of heterostructure electronic structure. At the interface, a shift in the average potentials occurs, which can be taken as the reference energy. The bulk band structures of the two component materials are then aligned according to these average potential positions. Consequently, a usual way to calculate the SBH is to use the macroscopic average potential as the referenced energy levels^29^. Meanwhile, a supercell calculation is performed in which the planar microscopic potential along the direction perpendicular to the interface (*z* axis in Fig. 1) is calculated in the heterostructure, by averaging the Hartree potentials in the plane parallel to the interface. Thus all the intrinsic interface effects, such as the chemical composition, structural distortion, orientation and strain are reflected in the potential.

In detail, the p-type SBH (*ϕ*_*p*_) of a GR/MO interface is obtained from the following procedure: (1) the difference (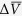) between the averaged local potentials of GR and MO is calculated in the heterostructure; (2) the Fermi level of GR 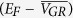 and the valence band edge level of MO 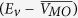 with respect to the averaged local potentials are calculated for the individual bulks of GR (i.e., graphite) and MO. The SBH is finally determined by:


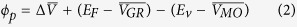


**Figure 4:**
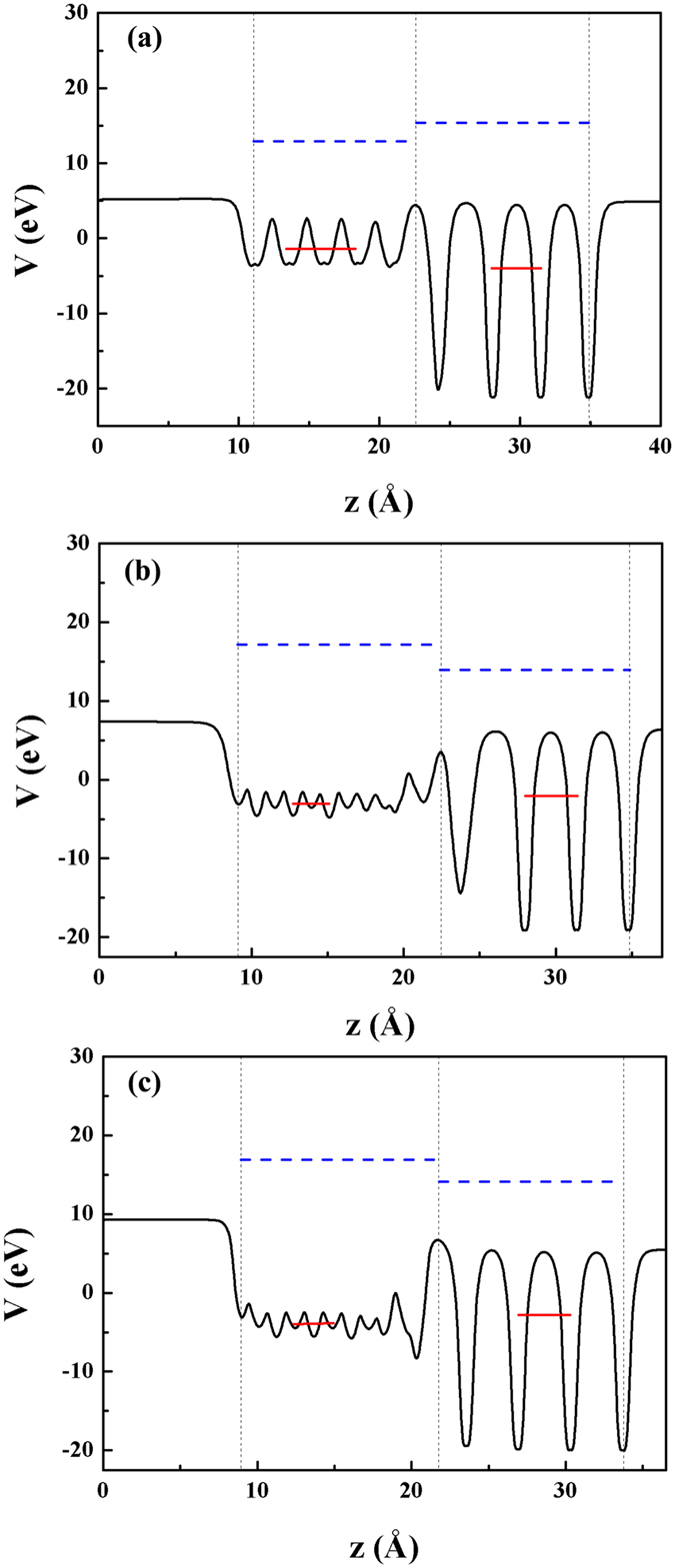
The black lines are planer-averaged local potentials for the (**a**) GR/Cu_2_O-O; (**b**) GR/NiO-Ni; and (**c**) GR/NiO-O. Vertical dashed lines represent the interface position (the middle one) and surfaces (the two side ones). The red solid lines are the average value of planer-averaged local potentials in the heterojunction, and the Fermi level of GR and the valence band maximum of MOs with respect to the averaged local potential are denoted by the horizontal blue dashed lines.

We average the planar-averaged local potential in the center bulk region of each material as 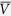, assuring that the internal fields caused by the interface dipoles in the supercell are removed. The planar-averaged local potential 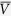 along *z* axis perpendicular to the interface is plotted in Fig. 4. Eventually, the calculated SBH is −0.414 eV for GR/NiO-Ni, −0.401 eV for GR/Cu_2_O-O, and −0.307 eV for GR/NiO-O interface, respectively. The negative value means that the Fermi level of GR locates above the VBM level of both MOs before matching, which is further supported by an independent calculation of the work functions of graphite, Cu_2_O and NiO surfaces and will be discussed below. Previously, similar SBH values were reported for other systems, e.g., 0.1 to 0.8 eV for MoS_2_/metal (Co, Ni, Cu, Mg)^47^, 0.37 eV for graphene/black phosphorene^48^, and 0.65 eV for GR/ZnO^49^. The small magnitude of SBH around 0.4 eV suggests a low contact resistance, and thus electrons can transport between these interfaces easily with little power loss.

To further elucidate the origin of SBH, we start from the Schottky-Mott limit^50^,^51^ which is based on frozen charge approximation at interface (i.e. when metal contact with semiconductor, the electrostatic potential obeys the superposition principle^27^). Under the condition that the charge rearrangement can be strictly avoided at the interface, the Schottky-Mott limit holds and yields the band alignment only through the individual bulk solids, i.e., the SBH at Schottky-Mott limit can be expressed as:


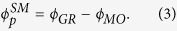


Here the *ϕ*_*MO*_ is the work function of MO; and *ϕ*_*GR*_ is the work function of graphite. From our calculations, the work function of graphite is 4.778 eV, consistent with the experimental value of 4.6 eV^52^. It is known polar NiO(111) surface can be stabilized by a particular p(2 × 2) “octopolar” reconstruction^53^,^54^. Based on the polar reconstruction surface, the calculated work function of NiO(111) surface is 5.801 eV, larger than the experimental value of about 5.3 eV^55^. The work function for Cu_2_O (111) surface is 5.003 eV. Then from equation (3), the Schottky-Mott limit are −1.023 eV for GR/NiO-Ni and GR/NiO-O interfaces, −0.233 eV for GR/Cu_2_O-O, respectively. For GR/Cu_2_O-O, it was found that *ϕ*_*p*_ is close to the Schottky-Mott limit with little charge transfer at the interface. For GR/NiO-O interface with 0.716 eV difference between *ϕ*_*p*_ and *ϕ*_*p*_^SM^, a big discontinuity of about 4 eV is found between the vacuum energy levels on the two sides of the interface, which can be related to the strong interface dipole. The GR/NiO-Ni interface with 0.609 eV difference between *ϕ*_*p*_ and *ϕ*_*p*_^SM^ also shows a big vacuum energy level discontinuity of about 2 eV. Accordingly, the strong interface dipole can be seen from the plane-averaged electron density difference in Fig. 2. From our calculations of the LDOS, the electronic state disturbances of the bottom layer of GR, which is caused by interfacial dipole, are minor in GR/Cu_2_O-O. However, for GR/NiO-Ni and GR/NiO-O, the electronic states are disturbed more strongly. In summary, the charge rearrangement at the interface region induces an interfacial dipole, resulting in the SBH deviated from Schottky-Mott limit. We expect that experimentalists pay attention to the SBH measurement of epitaxial GR/MO materials and reveal the origin of SBH.

## Conclusion

To briefly summarize, we theoretically explored several critical issues of GR/MO heterostructures for their applications as electrode of LIBs. It was found that GR interacts moderately with ‘Ni’ determinate NiO (111) face. The interaction between ‘O’ determinate Cu_2_O (111) and NiO (111) is much weak, and such a weak physical interaction make them retaining their own excellent electrochemical performance in their composites. Analysis of the electron densities further indicates an interfacial dipole produced in the interface. Using the macroscopically averaged electrostatic potential as reference, our first-principles calculations show that the SBH is below 1 eV for all three heterostructures, which means that these GR/MO electrode materials have low interfacial resistance. At last, by comparing the results from macroscopic average potential method and the Schottky-Mott limit, we found that the interfacial dipole plays a crucial role in determining the SBH.

## Additional Information

**How to cite this article**: Cheng, K. *et al*. Schottky barrier at graphene/metal oxide interfaces: insight from first-principles calculations. *Sci. Rep.* **7**, 41771; doi: 10.1038/srep41771 (2017).

**Publisher's note:** Springer Nature remains neutral with regard to jurisdictional claims in published maps and institutional affiliations.

## Supplementary Material

Supplementary Information

## Figures and Tables

**Table 1:** Supercell represents the heterostructure, (*n* × *n*)/(*m* × *m*) denotes that there are n MO units and m GR units in a supercell.

	GR/Cu_2_O-O	GR/NiO-Ni	GR/NiO-O
Supercell	(2 × 2)/(5 × 5)	(4 × 4)/(5 × 5)	(4 × 4)/(5 × 5)
Mismatch	1.8%	0.1%	0.1%
E_b_ (eV)	0.016	0.119	0.044
d_z_ (Å)	2.650	2.500	2.959
*ϕ*_*p*_ (eV)	−0.401	−0.414	−0.307
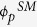 (eV)	−0.233	−1.023	−1.023

Lattice mismatch is defines as (*a*_*MO*_ − *a*_*GR*_)/*a*_MO_. We adopt the lattice parameters for MOs and slightly compress the graphene lattice to compensate the mismatch. Binding energy E_b_, equilibrium average distance d_z_, Schottky barrier height *ϕ*_*p*_, and Schottky barrier height at Schottky-Mott limit 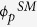 are for three heterostructures, respectively.
