# Supplementary material for: Schottky barrier at graphene/metal oxide interfaces: insight from first-principles calculations
Source: Sci Rep. 2017 Feb 6;7:41771. doi: 10.1038/srep41771 (PMC5292957; doi:10.1038/srep41771)
Supplement: Supplementary Information [file srep41771-s1.doc]

**Supporting information**

Schottky barrier at graphene/metal oxide interfaces: insight from first-principles calculations

Kai Cheng ^1^, Nannan Han ^1^, Yan Su ^1,*^, Junfeng Zhang^2^, Jijun Zhao^1^

^1^ Key Laboratory of Materials Modification by Laser, Ion and Electron Beams (Dalian University of Technology), Dalian 116024, P. R. China

^2^ School of physics and information Engineering (Shanxi Normal University), Linfen 041000, P. R. China

*Correspondence and requests for materials should be addressed to Y.S. (e-mail: su.yan@dlut.edu.cn)


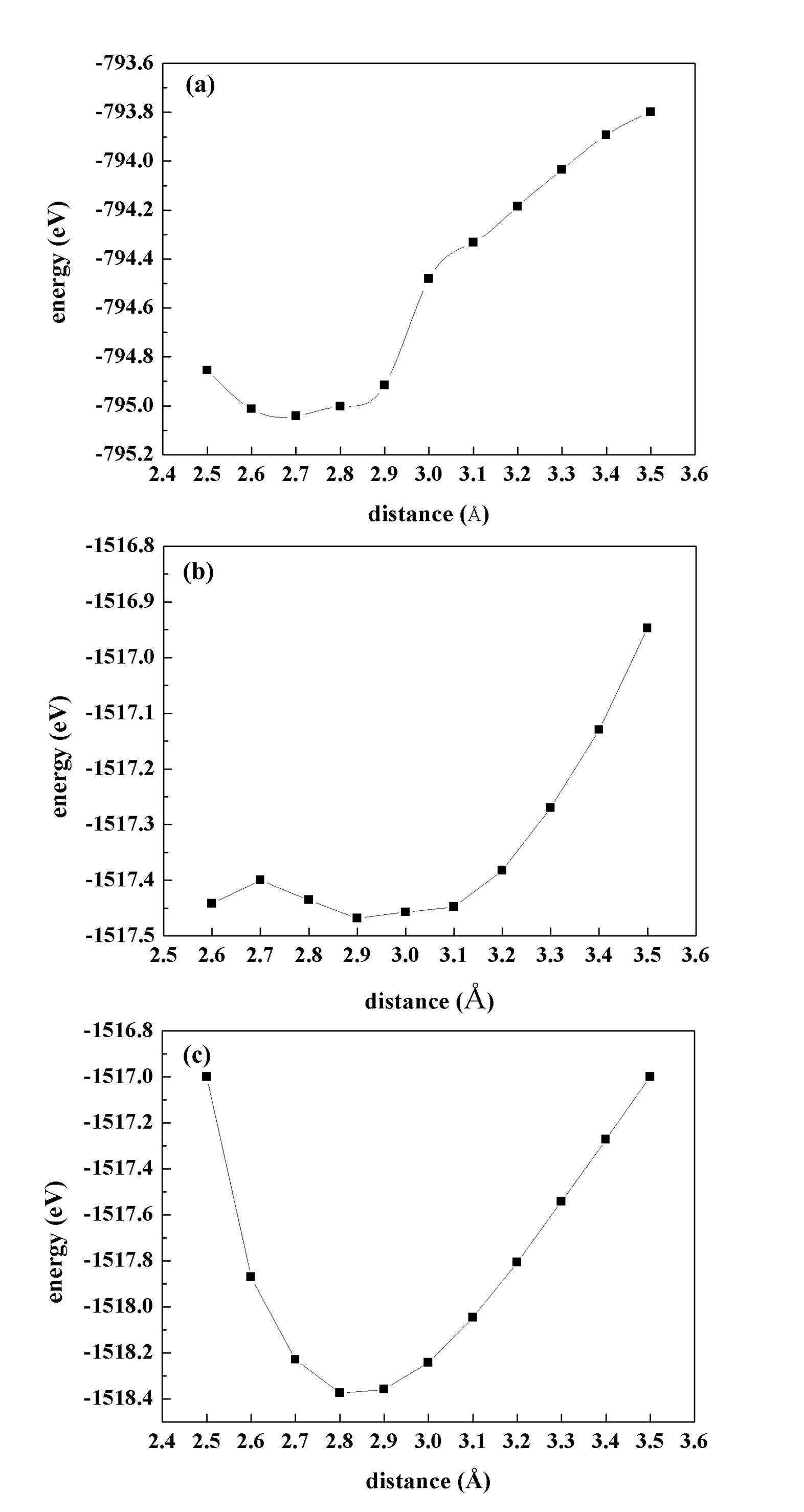


**Figure S1.** The single-point energy curves versus distances for (a) GR/Cu_2_O-O, (b) GR/NiO-Ni and (c) GR/NiO-O, respectively.
